# The Effect of Curcumin on Carbon Tetrachloride-Induced Lung Injury

**DOI:** 10.3390/biomedicines14081848

**Published:** 2026-08-17

**Authors:** Şahinaz Atalay, Mete Keçeci, Meryem Akpolat Ferah, Esra Babaoğlu

**Affiliations:** 1 Department of Histology and Embryology, Faculty of Medicine, Zonguldak Bülent Ecevit University, 67630 Zonguldak, Türkiye; atalay.sahinaz@gmail.com (Ş.A.); drmetehisto@gmail.com (M.K.); meryemakpolat@yahoo.com (M.A.F.); 2 Department of Anatomy, Faculty of Medicine, Zonguldak Bülent Ecevit University, 67100 Zonguldak, Türkiye

**Keywords:** carbon tetrachloride, curcumin, rat, lung, oxidative stress, inflammation

## Abstract

**Background/Objectives**: Carbon tetrachloride (CCl_4_) is a potent toxic agent that induces oxidative stress, inflammation, fibrosis, and apoptosis in various tissues, including the lungs. This study aimed to investigate the potential protective effects of curcumin against CCl_4_-induced lung injury in rats using histopathological, biochemical, and immunohistochemical analyses. **Methods**: A total of 40 male Wistar albino rats were allocated to four experimental groups: control, curcumin, CCl_4_, and CCl_4_ + Cur. Curcumin was administered orally at 200 mg/kg/day for three weeks, whereas CCl_4_ was administered intraperitoneally at 0.5 mL/kg as a 1:1 mixture with olive oil every other day for three weeks. **Results**: Histopathological examination revealed marked alveolar septal thickening, hemorrhage, vasocongestion, inflammatory cell infiltration, vacuolization, and epithelial desquamation in the CCl_4_ group, whereas lung tissue architecture was largely preserved in the CCl_4_ + Cur group. Biochemically, CCl_4_ exposure significantly increased the MDA levels and decreased SOD activity, while curcumin administration significantly reduced the MDA levels and increased SOD activity. Immunohistochemical H-score analysis showed significantly higher TNF-α, IL-1β, TGF-β, and caspase-3 immunoreactivity in the CCl_4_ group, whereas these alterations were significantly reduced following curcumin administration. **Conclusions**: These findings indicate that curcumin attenuated pulmonary histopathological, biochemical, and immunohistochemical alterations associated with CCl_4_ exposure, accompanied by improved oxidant–antioxidant balance and reduced inflammatory, profibrotic, and apoptotic immunoreactivity. However, because hepatic injury was not evaluated, the relative contributions of direct pulmonary effects and indirect liver-mediated systemic effects could not be determined.

## 1. Introduction

Carbon tetrachloride (CCl_4_) is a colorless, odorless, highly volatile chemical compound that has long been used as a solvent, cleaning agent, and chemical intermediate in industrial applications. Despite restrictions on its use in many countries, environmental and occupational exposure to CCl_4_ remains an important toxicological concern because of its harmful effects on multiple organs, including the liver, kidneys, brain, and lungs [1,2]. The primary routes of exposure to CCl_4_ are inhalation, dermal contact, and ingestion. Nevertheless, in experimental animal studies, CCl_4_-induced toxicity is commonly modeled by the intraperitoneal administration of CCl_4_ at a dose of 0.5 mL/kg [3]. Following exposure, CCl_4_ undergoes cytochrome P450-mediated metabolism, leading to the formation of highly reactive trichloromethyl (CCl_3_•) and trichloromethyl peroxyl (CCl_3_OO•) radicals [2]. These reactive intermediates initiate lipid peroxidation, oxidative stress, mitochondrial dysfunction, inflammation, and apoptosis, ultimately resulting in tissue injury [1,2].

Although the hepatotoxic effects of CCl_4_ have been extensively investigated, studies focusing on pulmonary injury remain relatively limited. The lungs are susceptible to ROS-mediated oxidative damage [4,5]. Excessive production of reactive oxygen species (ROS) in lung tissue promotes inflammatory cell infiltration, cytokine release, alveolar epithelial damage, vascular permeability, and fibrotic remodeling [4,5,6]. Previous experimental studies have demonstrated that CCl_4_ exposure induces the thickening of alveolar septa, inflammatory infiltration, hemorrhage, edema, fibrosis, and oxidative damage in lung tissue [6,7,8]. Furthermore, increased levels of pro-inflammatory mediators such as TNF-α, IL-1β, and TGF-β have been shown to contribute to pulmonary inflammation and fibrosis following CCl_4_ exposure [7,9].

Curcumin is a naturally occurring polyphenolic compound derived from the rhizome of Curcuma longa, commonly known as turmeric [10]. In recent years, curcumin has attracted considerable attention because of its broad therapeutic potential. In addition to its well-known antioxidant activity, curcumin exhibits anti-inflammatory, anti-apoptotic, antifibrotic, immunomodulatory, antimicrobial, and anticancer properties [10,11]. Experimental studies have demonstrated that curcumin suppresses ROS production, inhibits NF-κB signaling pathways, reduces pro-inflammatory cytokine release, attenuates mitochondrial dysfunction and reduces tissue fibrosis [9,10,11]. Furthermore, curcumin has shown protective effects in experimental models of lung injury associated with copper exposure and sodium nitrite-induced hypoxia, accompanied by modulation of the oxidative and inflammatory pathways [11,12].

Although CCl_4_-induced pulmonary oxidative stress, inflammation, and histopathological injury have been documented in experimental animal models [6,7,8], evidence regarding curcumin as a protective intervention in this setting remains limited. Accordingly, the contribution of the present study lies in the integrated histopathological, biochemical, and immunohistochemical evaluation of curcumin in an established model of CCl_4_-induced lung injury. Therefore, this study aimed to assess the potential effects of curcumin on CCl_4_-associated pulmonary alterations, with particular emphasis on lung tissue morphology, oxidant–antioxidant balance, and immunohistochemical markers associated with inflammatory, profibrotic, and apoptotic responses.

## 2. Materials and Methods

### 2.1. Chemicals and Reagents

Curcumin (Sigma-Aldrich, C1386) and olive oil (Sigma-Aldrich, 01514) used in the present study were purchased from Sigma-Aldrich (St. Louis, MO, USA). CCl_4_ was purchased from Saf Kimya (Istanbul, Türkiye). Commercial assay kits for the measurement of superoxide dismutase (SOD; RLD0123) and malondialdehyde (MDA; RLMD0158) in lung tissue were obtained from Rel Assay Diagnostics (Gaziantep, Türkiye).

### 2.2. Animals and Ethics

Male Wistar albino rats (*n* = 40; 8 weeks old; body weight, 300–350 g) were used in the present study. The rats were obtained from the Zonguldak Bülent Ecevit University Animal Care and Research Unit (Türkiye). Before the experiment, the animals were acclimatized to the laboratory environment for one week. During this period, they were handled daily by the investigators and weighed to minimize stress associated with handling and routine experimental procedures. All animals were healthy, experimentally naïve, and had not undergone any previous experimental procedures before inclusion in the study. During the experimental period, the animals were housed in standard plastic cages with stainless-steel wire lids and coarse wood-shaving bedding under controlled environmental conditions (20 ± 1 °C, 60 ± 10% humidity, and a 12 h/12 h light/dark cycle). The animals had ad libitum access to standard pelleted laboratory chow and tap water supplied in 500 mL plastic drinking bottles. Throughout the study, the Guide for the Care and Use of Laboratory Animals published by the U.S. Public Health Service was followed. The experimental protocol was approved by the Institutional Animal Ethics Committee of Bülent Ecevit University (Zonguldak, Türkiye; protocol no. 2022-01-06/01).

An a priori sample size calculation was not performed. Instead, the sample size was determined during the animal ethics committee application process based on previous experimental studies using similar animal models, in which 8–10 animals per group were commonly employed. Accordingly, ten animals were included in each experimental group.

Throughout the experimental period, all animals were monitored daily for general health and welfare. All experimental procedures were performed with the aim of minimizing pain, distress, and suffering throughout the study. No formal a priori inclusion or exclusion criteria were established before the study. Animals would have been excluded only if unexpected health problems or conditions compromising the experimental protocol had occurred; however, no such events were observed. Consequently, no animals, tissue samples, experimental units, or data points were excluded from the analyses. All analyses were performed using samples obtained from all animals (*n* = 10 per group).

### 2.3. Experimental Design

Formal randomization was not performed for group allocation. Prior to the experiment, all rats were weighed, and animals with comparable baseline body weights were allocated to the experimental groups to achieve a balanced baseline body weight distribution among the groups, thereby minimizing potential confounding related to baseline body weight differences. Each individual rat was considered one experimental unit throughout the study. A total of four groups were formed, comprising one control group and three experimental groups, with ten rats in each group. The control group received 1 mL of olive oil by oral gavage once daily for three weeks. Curcumin was administered at 200 mg/kg by oral gavage once daily for three weeks; the selected dose was based on a previously published rat study [13]. Curcumin was dissolved in olive oil for administration. The CCl_4_ group received 0.5 mL/kg of CCl_4_ dissolved in olive oil at a ratio of 1:1 via intraperitoneal (IP) injection every other day for three weeks [14]. Although occupational exposure to CCl_4_ occurs primarily through inhalation, with additional dermal and oral routes, experimental animal studies most commonly employ intraperitoneal administration to achieve a controlled and reproducible toxic exposure. Although intraperitoneal CCl_4_ administration has been widely used in experimental models of hepatotoxicity, it has also been validated as a reproducible model of pulmonary injury secondary to systemic oxidative stress and has been used to investigate the mechanisms of CCl_4_-induced lung injury and evaluate potential therapeutic interventions [2,7,15]. The CCl_4_ + Cur group received 200 mg/kg curcumin dissolved in olive oil via gavage on a daily basis and 0.5 mL/kg CCl_4_ dissolved in olive oil at a ratio of 1:1 via i.p injection for a period of three weeks.

At the end of the study, the animals were deeply anesthetized with intraperitoneal xylazine (10 mg/kg; Rompun, Bayer, Leverkusen, Germany) and ketamine (90 mg/kg; Ketalar, Eczacıbaşı, İstanbul, Türkiye) and subsequently euthanized by exsanguination. The right and left lungs were then excised, and the left lung was fixed in 10% formalin.

Group allocation was known during animal allocation and treatment administration. Histopathological, biochemical, immunohistochemical, and statistical analyses were performed by investigators who were blinded to the experimental group allocation.

The study outcomes included histopathological evaluation, biochemical assessment of tissue MDA and SOD levels, and semiquantitative immunohistochemical assessment of caspase-3, IL-1β, TNF-α, and TGF-β immunoreactivity.

The treatment doses, routes of administration, and experimental duration were selected based on previously validated experimental protocols.

### 2.4. Histopathological Evaluations and Scoring of Lung Damage

Paraffin blocks were prepared from left lung tissues following overnight fixation in 10% formalin. Sections were stained with hematoxylin and eosin (H&E), periodic acid–Schiff (PAS), and Masson’s trichrome to evaluate general lung histology, goblet cells, and connective tissue, respectively. All staining procedures were performed as described by Suvarna et al. [16]. Histological lung injury was assessed in H&E-stained sections. Ten randomly selected fields from each section were scored from 0 to 3 for vascular congestion, hemorrhage, alveolar septal thickening, vacuolization, macrophage accumulation, inflammatory cell infiltration, and epithelial desquamation. The scoring system was defined as follows: 0 = no damage, 1 = mild, 2 = moderate, and 3 = severe [15].

All histological assessments were performed using an Axio Lab.A1 light microscope (Zeiss, Oberkochen, Germany) with AxioVision Rel. 4.8 software at ×200 magnification.

### 2.5. Evaluation of SOD and MDA Levels

Right lung tissues were used for biochemical analyses. Tissue SOD and MDA levels were measured using commercially available assay kits according to the manufacturer’s instructions [17,18]. Samples were stored at −80 °C until analysis.

### 2.6. Immunohistochemical Evaluations

Immunoreactivity for caspase-3, IL-1β, TNF-α, and TGF-β was evaluated using a semiquantitative immunohistochemical H-score system.

For immunohistochemical detection, sections of 5 µm thickness, prepared from paraffin blocks, were mounted on positively charged slides. Following deparaffinization and immersion, the sections in citrate buffer were maintained at a temperature just below the boiling point for 15 min in a microwave oven, thereby facilitating the unmasking of the antigenic binding sites. Subsequently, the sections were placed in distilled water and permitted to cool at room temperature for a period of 20 min. Following three washes with PBS, the sections were treated with 3% H_2_O_2_ for 10 min to block endogenous peroxidase activity. Following a seven-minute incubation period with Ultra V block (LabVision, TA-015-UB, Utrecht, The Netherlands) to prevent non-specific binding, the sections were treated with primary antibodies against caspase-3 (Thermo Fisher Scientific, Waltham, MA, USA), IL1-β (Novus Biologicals, Centennial, CO, USA), TGF-β (Thermo Fisher Scientific, Waltham, MA, USA), and TNF-α (Abcam, Cambridge, UK) for a period of 24 h at a temperature of +4 °C. The subsequent step involved the application of the secondary antibody (biotinylated link, Dako, K0609, Glostrup, Denmark) and streptavidin-peroxidase (streptavidin HRP, Dako, K0609, Glostrup, Denmark) for 30 and 10 min, respectively, at room temperature, following a wash with PBS. Subsequently, the sections were treated with a diaminobenzidine (DAB) chromogen solution (Vector, SK-4100, Newark, CA, USA) until the optimal staining intensity was achieved under a light microscope. Thereafter, the sections were counterstained with hematoxylin. Throughout the course of all incubations, the tissues were maintained in a humid environment in order to prevent the tissues from drying and resulting in background staining. The sections that had been closed using Entellan and a coverslip were examined under a light microscope (Carl Zeiss Microscopy GmbH, Jena, Germany). The findings were photographed using a Zeiss Axio Lab. A1 brand photomicroscope (Carl Zeiss Microscopy GmbH, Jena, Germany). All immunohistochemical analyses were performed in accordance with the manufacturer’s recommended protocols.

The histological scoring (H-score) was conducted manually in accordance with the established criteria for defining immunohistochemical results. These criteria entail the following gradations: 0, no staining; 1+, weak but discernible staining; 2+, medium or pronounced staining; and 3+, intense staining. The H-score value for each section was calculated by multiplying the percentage of stained cells within each density category by the corresponding density. The scoring was conducted under a light microscope at ×40 objective magnification on 20 randomly selected fields on each section, and the mean scores were employed for statistical analysis. H-score = Σ(i × Pi), i; intensity score, Pi; percentage of positively stained cells [19].

### 2.7. Statistical Analysis

The statistical evaluation was conducted using the Jamovi Desktop 2.3.21 software. Data distribution was assessed using the Kolmogorov–Smirnov test. Data satisfying the assumptions of normality were analyzed using parametric tests, whereas non-normally distributed data were analyzed using the corresponding non-parametric tests. Descriptive statistics for data that were normally distributed data were analyzed using one-way analysis of variance (ANOVA), followed by Tukey’s post hoc test. Non-normally distributed data were analyzed using the Kruskal–Wallis test, followed by Dunn’s multiple-comparison test where appropriate. A *p*-value < 0.05 was considered statistically significant.

## 3. Results

### 3.1. Biochemical Findings

**Figure 1:**
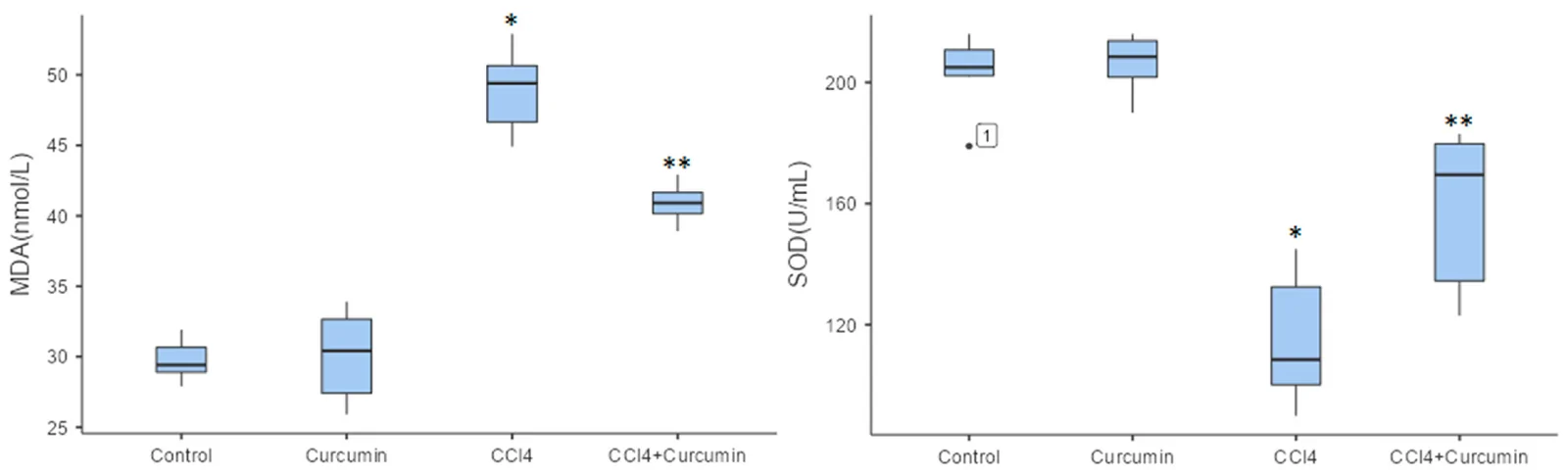
Comparison of MDA levels and SOD activity in lung tissues among the experimental groups. CCl_4_ administration significantly increased the MDA levels and decreased SOD activity compared with the control and curcumin groups, whereas curcumin administration significantly reduced the MDA levels and increased SOD activity in the CCl_4_ + Cur group. Data are presented as median (minimum–maximum). * *p* < 0.01 vs. control, curcumin, and CCl_4_ + Cur groups; ** *p* < 0.01 vs. control, curcumin, and CCl_4_ groups. The number 1 indicates the case number of the outlier observation.

MDA levels were significantly lower in the CCl_4_ + Cur group than in the CCl_4_ group, whereas SOD activity was significantly higher in the CCl_4_ + Cur group than in the CCl_4_ group (*p* < 0.01 for both comparisons; Figure 1).

### 3.2. Histopathological Findings

**Figure 2:**
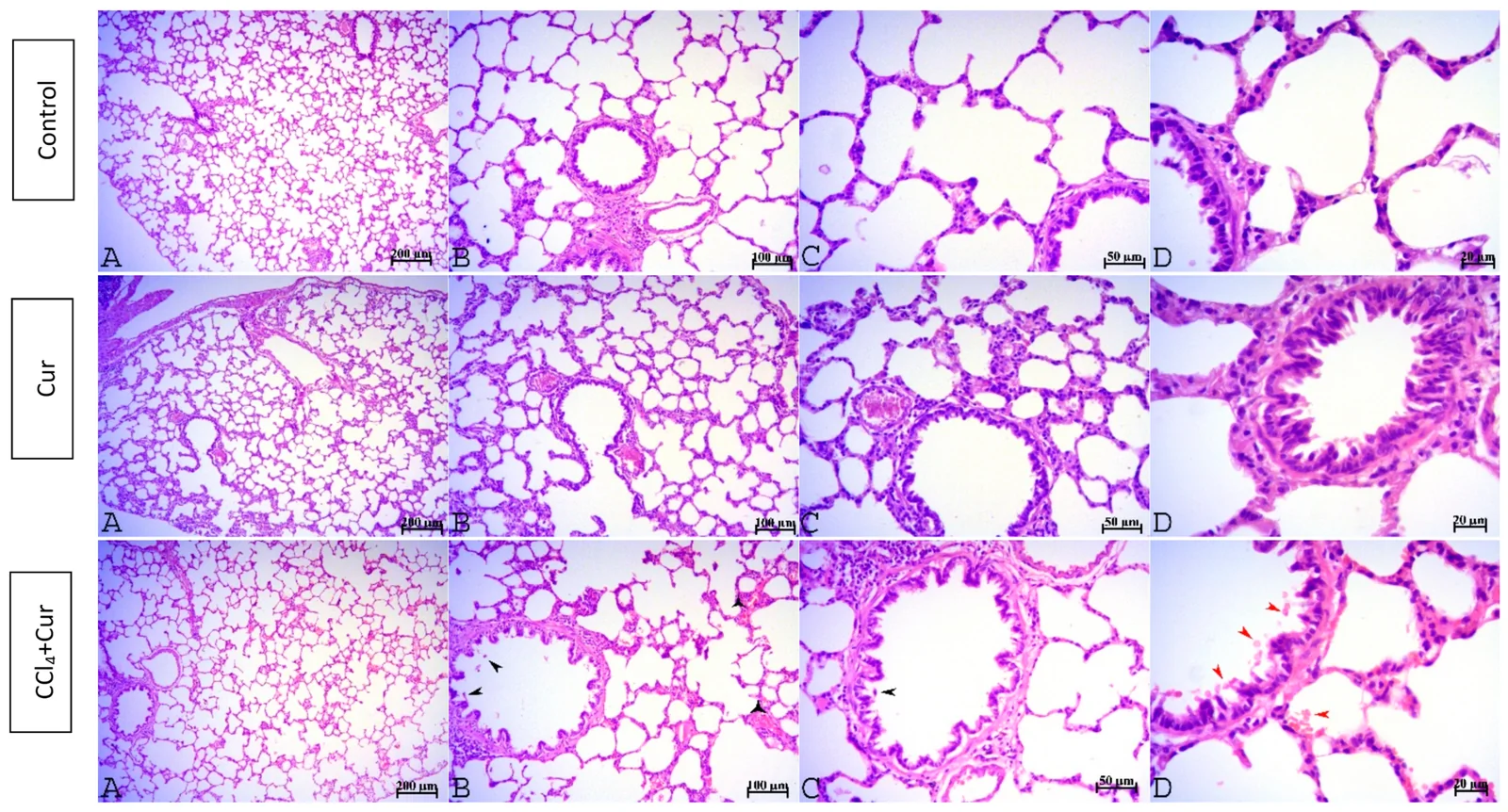
Representative H&E-stained lung sections from the control, curcumin, and CCl_4_ + Cur groups. For each experimental group, panels A–D represent the respective histological images at different magnifications. Limited areas of bronchial epithelial desquamation (black arrowhead), hemorrhage within the alveolar and bronchiolar lumens (red arrowhead), and vascular congestion (three-armed black star) are indicated in the CCl_4_ + Cur group. H&E staining. Scale bars: (**A**) 200 µm; (**B**) 100 µm; (**C**) 50 µm; (**D**) 20 µm.

**Figure 3:**
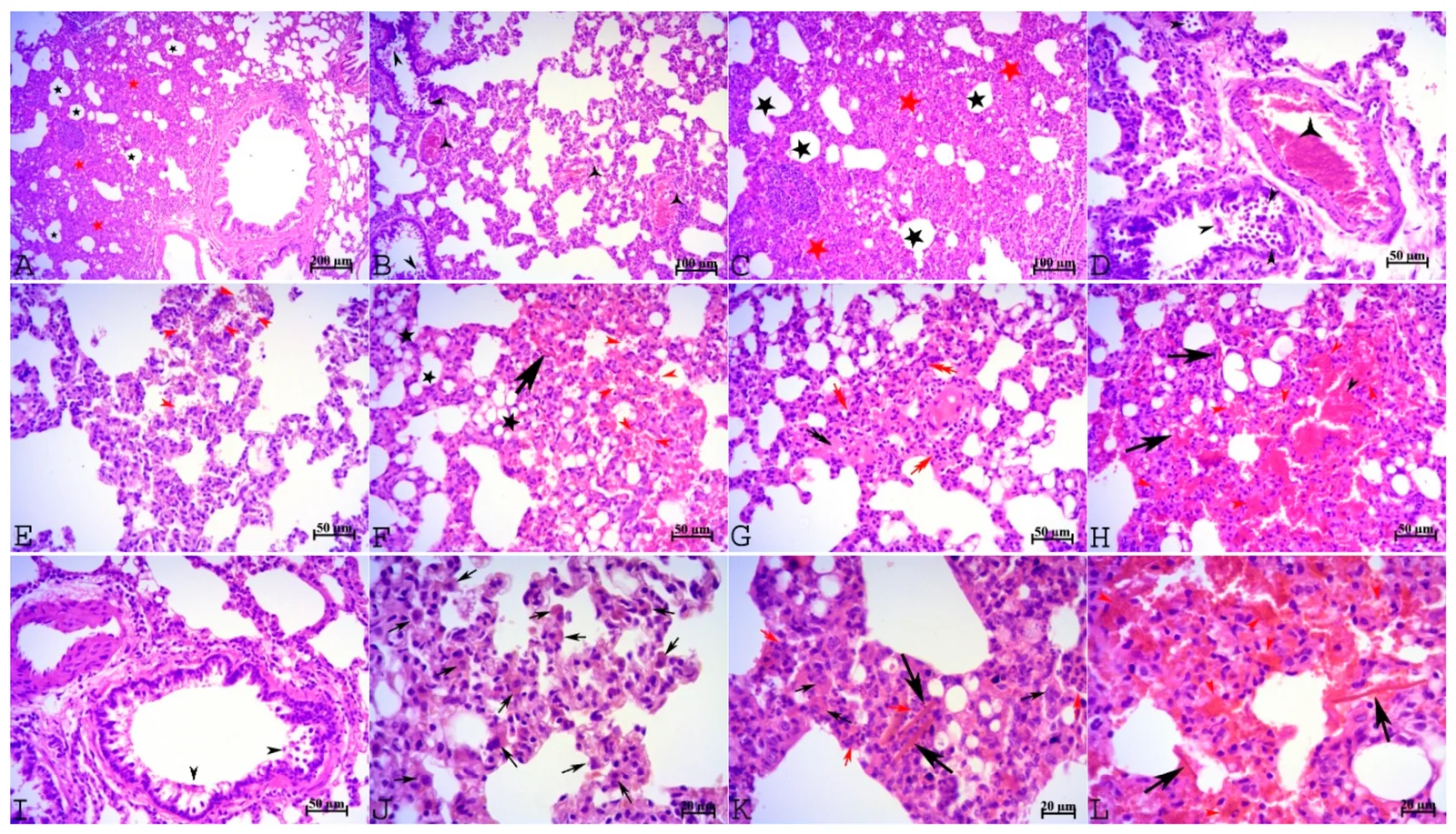
Representative H&E-stained lung sections from the CCl_4_ group. Alveolar septal thickening (red star), vacuolization (black star), epithelial desquamation (black arrowhead), vascular congestion (three-armed black star), alveolar and septal hemorrhage (red arrowhead), erythrocyte crystals (thick black arrow), alveolar and septal macrophages (thin black arrow), neutrophilic infiltration within the alveolar septa (thin red arrow), focal alveolar septal edema (double-headed black arrow), and lymphocytic infiltration within the alveolar septa (red double-headed arrow). H&E staining. Scale bars: (**A**) 200 µm; (**B**,**C**) 100 µm; (**D**–**I**) 50 µm; (**J**–**L**) 20 µm.

Histopathological changes in lung tissues from all groups were evaluated using H&E, Masson’s trichrome, and PAS staining. In the H&E-stained sections, lung tissues from the control and curcumin groups exhibited normal histological architecture (Figure 2). In the CCl_4_ group, marked thickening of the interalveolar septa resulted in a more compact appearance of the lung parenchyma, accompanied by prominent vacuolization. Dense neutrophilic infiltration, alveolar macrophages, and areas of lymphocytic infiltration were also observed. In addition, pronounced hemorrhage and vascular congestion were present. Rod-shaped erythrocyte crystals were observed within areas of hemorrhage. Focal edema was detected in only one animal in the CCl_4_ group (Figure 3). In the CCl_4_ + Cur group, bronchial epithelial desquamation (black arrowhead), hemorrhage in the alveolar and bronchiolar lumens (red arrowhead), and vascular congestion (three-armed black star) were observed only in limited areas, whereas the overall lung architecture was largely similar to that of the control and curcumin groups (Figure 2). Histopathological scores differed significantly among the groups for all evaluated parameters, as shown in Table 1.

**Figure 4:**
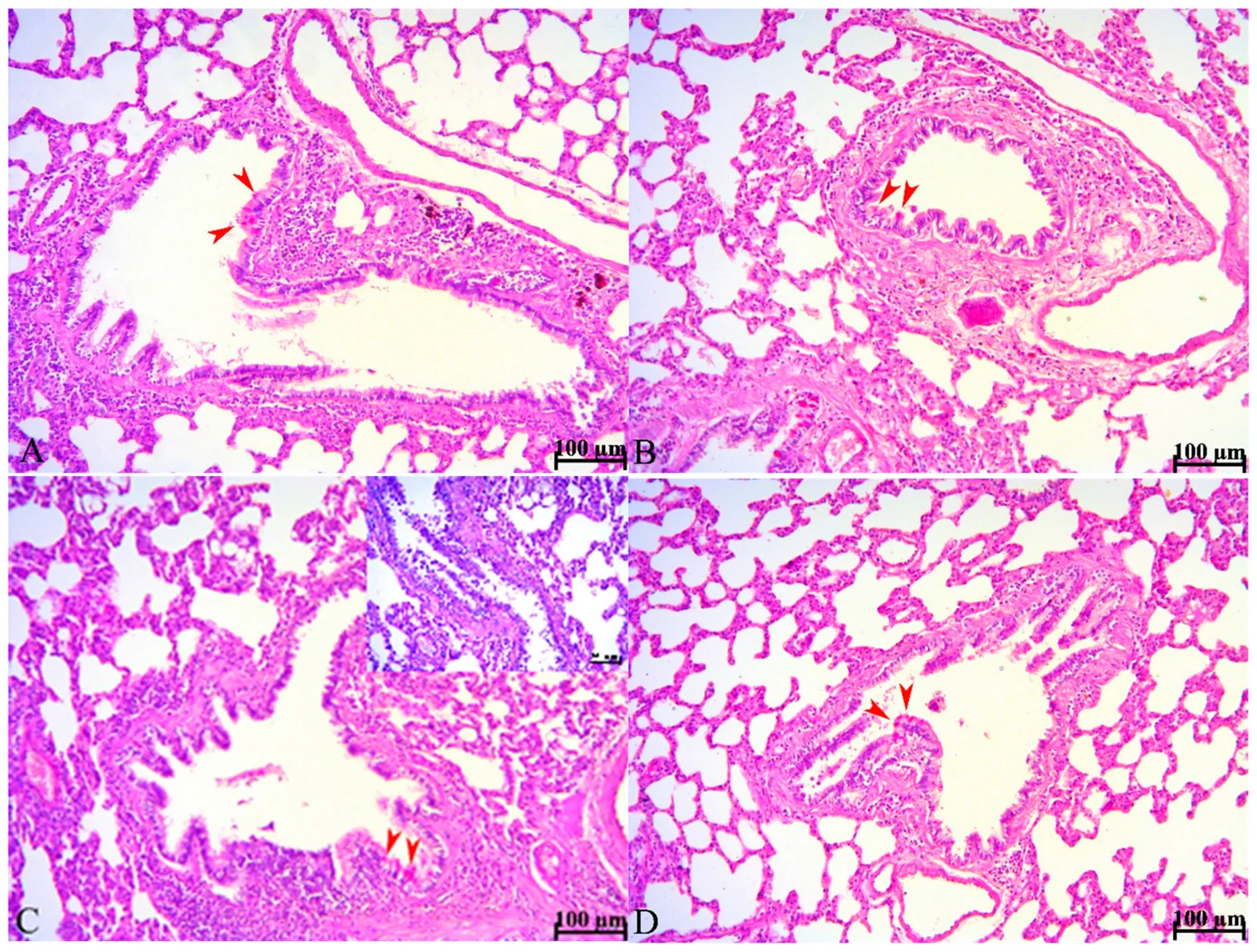
Representative PAS-stained lung sections from all experimental groups. Goblet cells in the bronchiolar epithelium are indicated by red arrowheads. Loss of goblet cells associated with epithelial desquamation is shown in the CCl_4_ group (inset). (**A**) Control; (**B**) curcumin; (**C**) CCl_4_; (**D**) CCl_4_ + Cur. PAS staining. Scale bar: 100 µm; inset scale bar: 50 µm.

PAS-stained lung sections showed a similar distribution of bronchiolar goblet cells among the experimental groups. In the CCl_4_ group, however, goblet-cell loss was observed in areas associated with bronchial epithelial desquamation (Figure 4).

**Figure 5:**
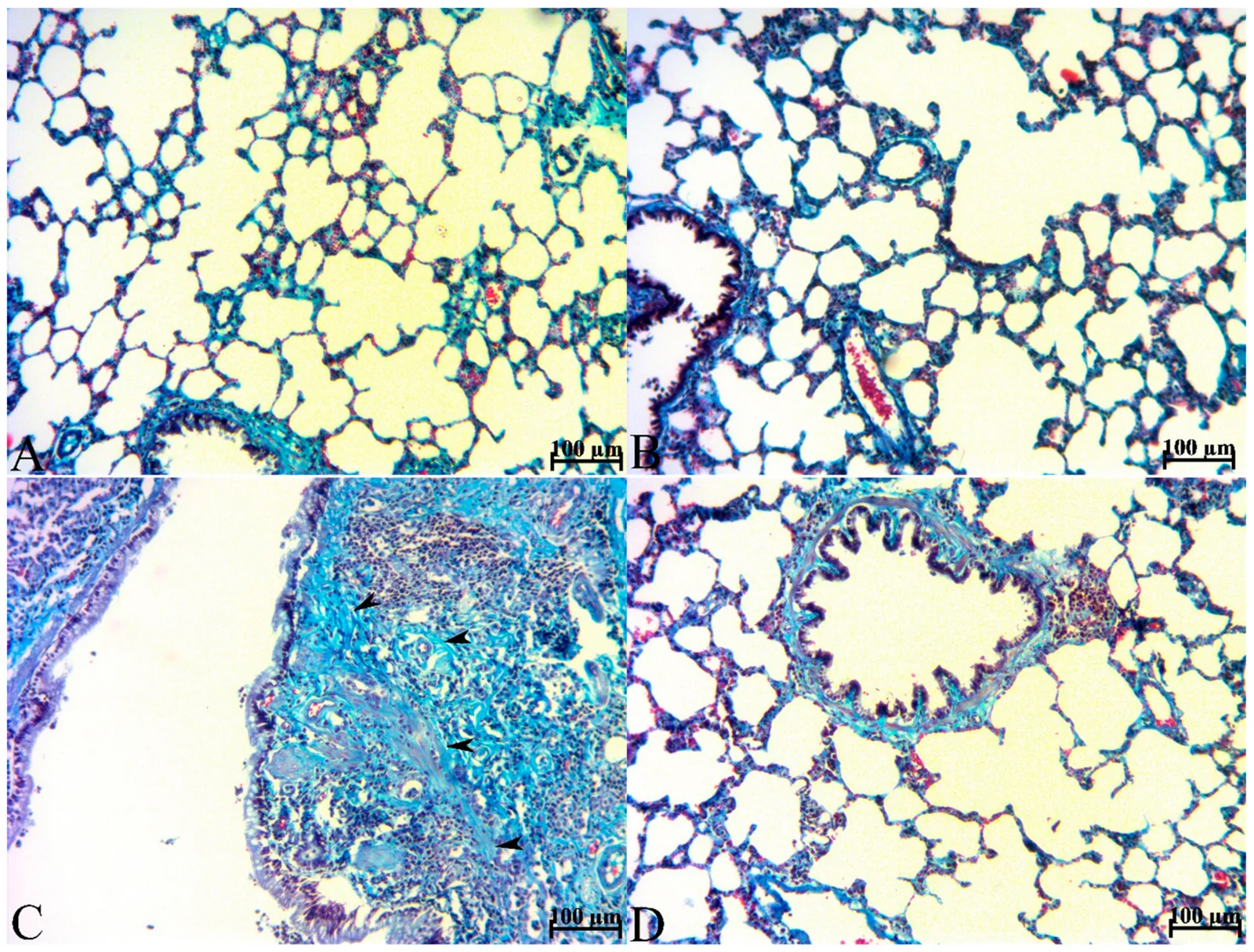
Representative Masson’s trichrome-stained lung sections from the experimental groups. The CCl_4_ group shows thickened interalveolar septa and increased peribronchiolar collagen deposition, whereas these alterations are markedly attenuated in the CCl_4_ + Cur group. Thick collagen bundles in the peribronchiolar region are indicated by the black arrowhead. (**A**) Control; (**B**) curcumin; (**C**) CCl_4_; (**D**) CCl_4_ + Cur. Masson’s trichrome staining. Scale bar: 100 µm.

Masson’s trichrome staining showed thin interalveolar septa in the control and curcumin groups. In the CCl_4_ group, interalveolar septal thickening was accompanied by inflammatory cell infiltration and increased peribronchiolar connective tissue deposition. Although septal thickening associated with inflammatory cell infiltration was also observed in the CCl_4_ + Cur group, no marked increase in peribronchiolar connective tissue was detected (Figure 5).

### 3.3. Immunohistochemical Findings

**Figure 6:**
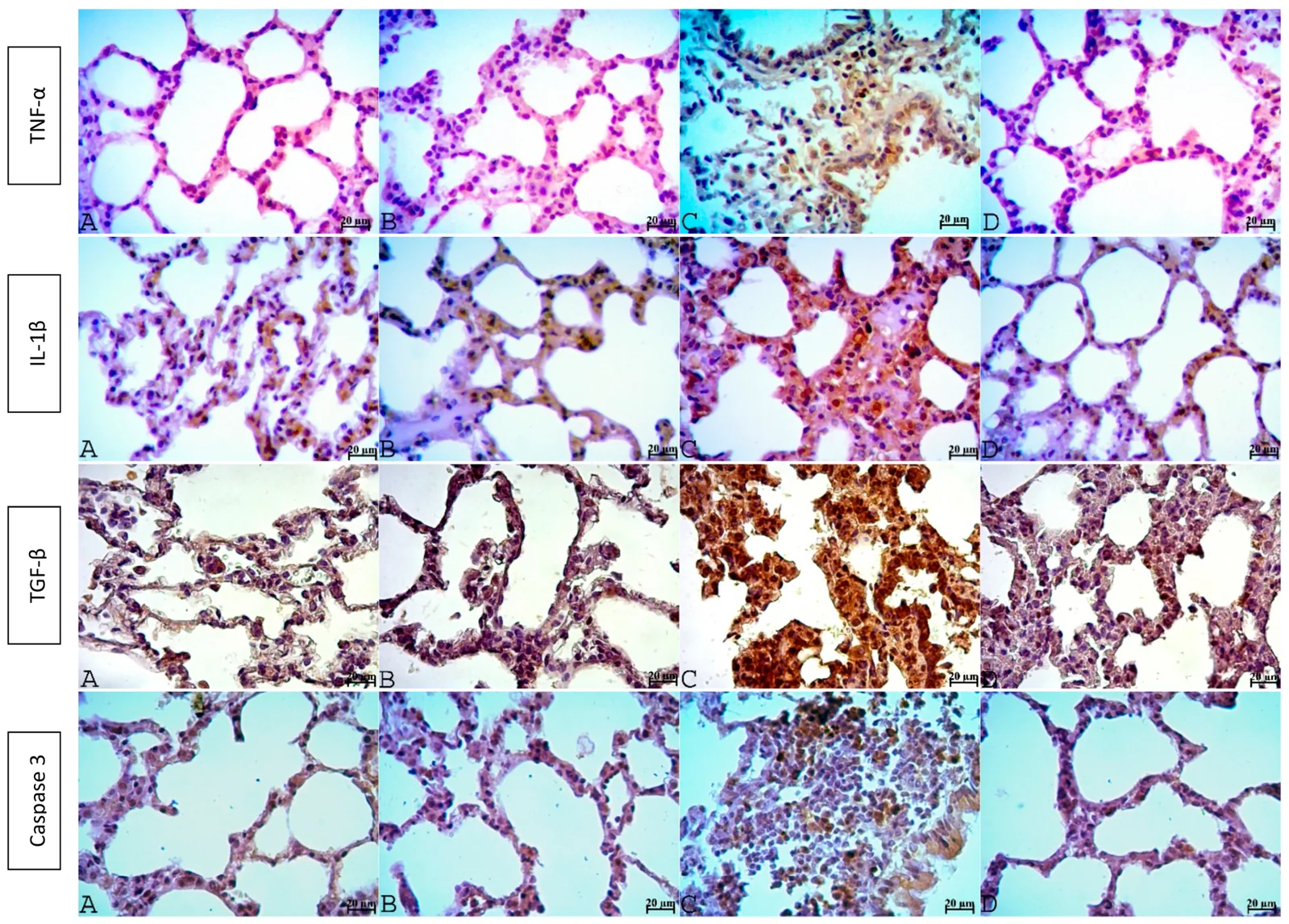
Representative immunohistochemical staining of TNF-α, IL-1β, caspase-3, and TGF-β in lung tissues from the experimental groups. For each experimental group, panels A–D represent the respective histological images at different magnifications. Immunoreactivity for TNF-α, IL-1β, caspase-3, and TGF-β was higher in the CCl_4_ group and was reduced in the CCl_4_ + Cur group. (**A**) Control; (**B**) curcumin; (**C**) CCl_4_; (**D**) CCl_4_ + Cur. Immunohistochemical staining. Scale bar: 20 µm.

Immunohistochemical H-score analysis showed significantly higher TNF-α, IL-1β, TGF-β, and caspase-3 immunoreactivity in the CCl_4_ group than in the control, curcumin, and CCl_4_ + Cur groups (*p* < 0.05 for all comparisons; Figure 6 and Table 2). TGF-β immunoreactivity in the CCl_4_ + Cur group remained significantly higher than that in the control and curcumin groups (*p* < 0.05), whereas the other evaluated markers did not show this pattern.

## 4. Discussion

Previous experimental studies have established that CCl_4_ exposure can induce pulmonary oxidative stress, inflammation, and histopathological injury [6,7,8]. Therefore, the contribution of the present study does not lie in demonstrating CCl_4_-induced lung injury itself, but in evaluating curcumin as a protective intervention in this established model through an integrated assessment of lung histopathology, tissue MDA and SOD levels, and immunohistochemical markers associated with inflammation, fibrotic remodeling, and apoptosis. The findings provide complementary evidence that curcumin administration is associated with attenuation of these pulmonary alterations following CCl_4_ exposure.

The lungs are particularly vulnerable to damage caused by reactive oxygen species (ROS). Oxidative stress can result in acute inflammatory lung injury, which may subsequently lead to fibrotic remodeling and functional impairment [4]. Oxidative stress can be initiated or amplified by inflammation, hypoxia, hyperoxia, and drug exposure [20].

The toxicity of CCl_4_ has been attributed to the production of free radical derivatives [1]. Increased levels of ROS lead to the infiltration of inflammatory cells and the intense secretion of cytokines such as TNF-α, IL-1β, IL-6, and TGF-β [5,6,7]. Intraperitoneal CCl_4_ administration in rats has been demonstrated to induce alterations in the structure of the alveoli in lung tissue, marked thickening of the alveolar septa, an increase in inflammatory cells, congestion, edema, vacuolization, epithelial desquamation, an increase in alveolar macrophages, and, in some cases, severe pulmonary fibrosis. These alterations have been associated with oxidative stress [7,8,15]. The histopathological findings observed in the CCl_4_ group in our study were consistent with the literature. In the present study, the presence of erythrocyte crystals was observed in areas of congestion and hemorrhage in H&E-stained lung tissues of subjects exposed to CCl_4_. In the study conducted by Pääkkö et al., which examined intraalveolar hemorrhage areas by light microscopy following acute CCl_4_ exposure, the formation of crystallized hemoglobin was identified. This formation was defined as the immunoreactive staining of intraalveolar crystalline particles with anti-hemoglobin antibody. An important consideration in interpreting these findings is whether the observed pulmonary alterations and their attenuation by curcumin reflect local effects within the lung or systemic consequences of hepatic injury. CCl_4_-induced hepatic injury and fibrosis are strongly dependent on CYP2E1-mediated bioactivation [21]. Curcumin has also been reported to attenuate CCl_4_-induced hepatic oxidative stress, inflammation, apoptosis-related activity, and TGF-β1/Smad3 signaling [22]. Therefore, reduced hepatic injury and the consequent attenuation of circulating inflammatory and oxidative mediators may have contributed to the pulmonary improvements observed in the CCl_4_ + Cur group. Conversely, pulmonary club cells possess cytochrome P450-dependent metabolic activity capable of locally bioactivating CCl_4_, supporting the possibility of a direct pulmonary component [23]. Moreover, pulmonary vascular remodeling and collagen deposition have been reported in chronic CCl_4_-induced cirrhotic models, illustrating a potential liver–lung interaction [24]. Thus, the pulmonary findings observed in the present study may reflect direct pulmonary effects, indirect liver-mediated systemic effects, or a combination of both. Because neither liver function parameters nor hepatic histopathology were evaluated, the relative contributions of these mechanisms cannot be determined from the present experimental design.

It has been observed that there is an increase in interstitial connective tissue in Masson’s trichrome-stained lung tissue sections caused by the presence of CCl_4_. In a study by Das et al., the accumulation of extracellular matrix in peribronchiolar and perivascular adventitia was demonstrated in the lung tissues of mice with CCl_4_-induced liver cirrhosis. Das et al. demonstrated extracellular matrix accumulation in the peribronchial and perivascular adventitia, together with collagen deposition in the vascular media, adventitia, and lung parenchyma of CCl_4_-induced cirrhotic mice [24]. Similarly, CCl_4_-induced pulmonary oxidative damage and fibrotic alterations have been reported in Sprague–Dawley rats [25]. In the present study, increased peribronchiolar connective tissue deposition was observed in the CCl_4_ group, whereas no marked increase in peribronchiolar connective tissue was detected in the CCl_4_ + Cur group. These findings are consistent with attenuation of the oxidative stress–inflammation–fibrogenesis axis following curcumin administration.

In murine models of chronic asthma induced by ovalbumin, an increase in the number of goblet cells in areas of inflammation has been reported [26,27]. However, a paucity of research has been identified that examined the impact of CC_l4_ exposure on bronchial and/or bronchiolar goblet cells. In our study, the airways exhibited similar characteristics in all groups in terms of goblet cells, except for the areas of epithelial shedding encountered in the CCl_4_ group. These findings indicate that marked goblet-cell alterations were not a prominent feature of the present CCl_4_-induced lung injury model, except in areas of epithelial desquamation.

The release of free radicals, a by-product of CCl_4_ metabolism, serves as a catalyst for the initiation of lipid peroxidation, a process that involves the oxidation of polyunsaturated fatty acids within the cell membrane. This results in an increase in the levels of MDA, the final product of lipid peroxidation [28]. Increased lipid peroxidation and MDA levels are associated with sustained oxidative stress and pulmonary tissue injury following CCl_4_ exposure [29,30]. In the present study, the highest MDA levels were observed in the CCl_4_ group, while a significant decrease in MDA levels was observed in the group treated with curcumin in conjunction with CCl_4_. Previous studies have demonstrated a substantial decline in pulmonary SOD activity following CCl_4_ exposure [29,31]. In the present study, the levels of superoxide dismutase (SOD) were found to be low in the CCl_4_ group, while SOD activity was observed to increase significantly in the CCl_4_ + Cur group. The observed changes in SOD and MDA are consistent with an improvement in oxidant–antioxidant balance following curcumin administration.

The protective effects associated with curcumin may involve several interconnected processes; however, these mechanisms are not unique to the present pulmonary model. Curcumin has previously been reported to exert antioxidant, anti-inflammatory, anti-apoptotic, and antifibrotic effects in CCl_4_-induced injury models involving other organs, particularly the liver [10,11,22]. In the present study, the decrease in MDA levels and the increase in SOD activity in the CCl_4_ + Cur group suggest the attenuation of lipid peroxidation and improvement of antioxidant defense. This observation is consistent with previous experimental lung injury studies demonstrating reduced oxidative stress markers and enhanced antioxidant enzyme activity following curcumin administration [11,12]. Similarly, the reduced TNF-α and IL-1β immunoreactivity in the CCl_4_ + Cur group may reflect the attenuation of ROS-associated inflammatory activity and may be related to the accompanying reduction in inflammatory cell infiltration, vascular congestion, and tissue injury [4,5,9]. The lower TGF-β immunoreactivity and the absence of marked peribronchiolar connective tissue deposition are also consistent with the attenuation of inflammation-associated profibrotic responses [32,33]. Furthermore, reduced caspase-3 immunoreactivity suggests a lower apoptosis-related response, potentially associated with the reduction in oxidative and inflammatory injury [5,34,35]. Nevertheless, these findings do not identify a novel molecular pathway or provide definitive evidence of pathway-specific inhibition. Furthermore, because the immunohistochemical findings were not corroborated by Western blotting or quantitative PCR, they should be interpreted as semiquantitative tissue-level evidence of altered immunoreactivity rather than as definitive confirmation of changes in total protein abundance, gene expression, or pathway activation. Rather, the specific contribution of the present study lies in the integrated demonstration of concordant histopathological, biochemical, and immunohistochemical alterations within the same CCl_4_-induced pulmonary injury model. Collectively, these lung-tissue findings are consistent with the attenuation of oxidative, inflammatory, profibrotic, and apoptosis-related responses following curcumin administration; however, they do not establish whether the observed effects resulted from direct pulmonary actions, the indirect attenuation of hepatic and systemic injury, or a combination of both.

An additional consideration is the limited oral bioavailability of conventional curcumin, which is related to low intestinal absorption and rapid metabolism [10,36]. Although oral administration of curcumin at 200 mg/kg was associated with significant pulmonary tissue alterations in the present study, this experimental dose cannot be directly extrapolated to conventional oral curcumin use in humans. Moreover, because the plasma and lung tissue concentrations of curcumin and its metabolites were not measured, the systemic exposure achieved in the experimental animals and its relationship to the observed pulmonary responses remain unknown. Human pharmacokinetic data demonstrate that systemic curcuminoid exposure is strongly formulation-dependent, with micellar and dried colloidal preparations producing greater dose-normalized exposure than a standard turmeric extract [36]. Therefore, the present findings should not be interpreted as evidence that comparable pulmonary effects would necessarily be achieved in humans using conventional oral curcumin formulations. Future studies should incorporate pharmacokinetic measurements, exposure–response analyses, and comparisons of conventional curcumin with bioavailability-enhanced formulations.

The relationship between high ROS levels and inflammation is explained through gene transcription. It has been demonstrated that ROS exerts a direct influence on a variety of transcription factors, including NF-Kβ, AP-1, and HIF-1. These transcription factors have the capacity to interact either directly or indirectly with specific DNA motifs present within the promoters of target genes. Furthermore, ROS has been observed to trigger MAPK, tyrosine kinase, and tyrosine phosphatase cascades, which in turn activate these transcription factors, thereby amplifying the expression of proinflammatory cytokines and mediators [5,9]. It has been established that exposure to CCl_4_ leads to an augmentation in mitochondrial-derived ROS, which subsequently elevates the activities of proinflammatory enzymes and cytokines, including COX-2, IL-1β, TNF-α, and IL-6 [5,34]. Increased ROS production within the cell via NADPH oxidase or the mitochondrial electron transport chain leads to dissociation of the thioredoxin-interacting protein (TXNIP) complex and the binding of TXNIP to NLRP3, a type of inflammasome, leading to the formation of the active inflammasome. The active inflammasome converts pro-interleukin-1 (IL-1) beta and pro-IL-18 into active IL-1 beta and IL-18 [5,9]. Alveolar and interstitial macrophages within the lungs function as immune effector cells, rapidly responding to both infectious and allergenic stimuli [37]. The accumulation of active macrophages at the site of inflammation can result in tissue damage when it is excessive and uncontrolled. Classically activated macrophages, which have been demonstrated to contribute to tissue damage, are considered a rich source of TNF-α in lung injury models. These cells have also been shown to be responsible for the release of IL-1β and IL-6 [5,38]. In the present study, TNF-α and IL-1β immunoreactivity was significantly higher in the CCl_4_ group and was accompanied by marked macrophage accumulation in lung tissue. In the CCl_4_ + Cur group, both macrophage accumulation and cytokine immunoreactivity were reduced.

TNF-α has been shown to increase TGF-β1 expression in lung fibroblasts through AP-1-dependent transcriptional regulation [33]. As demonstrated in the current literature, both TGF-β and IL-1β have receptors on keratocytes, corneal fibroblasts, and myofibroblasts. IL-1β has been shown to play a role in the transformation of keratocytes into fibroblasts during corneal damage. Furthermore, these fibroblasts are converted into myofibroblasts by TGF-β [39]. Transient overexpression of IL-1β in rat lungs by intratracheal adenoviral gene transfer induced acute pulmonary inflammation and tissue injury, followed by increased TGF-β1 levels and progressive pulmonary fibrosis [40]. In the present study, the increase in TGF-β, concomitant with the rise in proinflammatory cytokines, as evidenced by Masson’s trichrome staining of the lung tissues from the CCl_4_ group, is consistent with the fibrotic changes observed. The administration of curcumin led to a reduction in the immunoreactivity of these markers previously enumerated, as well as the fibrotic changes.

Elevated oxidative stress and inflammatory signaling can contribute to the activation of apoptotic pathways [35]. Adel et al. reported increased oxidative stress, inflammation, and apoptosis-related alterations in CCl_4_-induced lung injury [34]. In the present study, caspase-3 immunoreactivity was significantly higher in the CCl_4_ group and was reduced following curcumin administration. This finding is consistent with the accompanying reductions in oxidative stress and inflammatory alterations; however, because additional apoptosis-related proteins were not assessed, these data should be interpreted as evidence of altered caspase-3 immunoreactivity rather than definitive demonstration of a specific apoptotic pathway.

### 4.1. Study Limitations

This study has several limitations. First, the experiments were conducted only in male Wistar albino rats; therefore, caution should be exercised when extrapolating these findings to females or humans. Second, only a single dose and treatment duration of curcumin and CCl_4_ were evaluated. Third, neither serum liver function parameters nor hepatic histopathological findings were evaluated. Given that CCl_4_ is a potent hepatotoxic agent and that curcumin may attenuate CCl_4_-induced hepatic injury, the present design cannot determine whether the observed pulmonary improvements resulted from direct effects on lung tissue, indirect effects mediated by reduced hepatic injury and systemic inflammation, or both. Consequently, the findings should not be interpreted as establishing a lung-specific protective mechanism. Future studies should evaluate hepatic and pulmonary outcomes concurrently, together with circulating inflammatory and oxidative stress markers, to clarify the relative contributions of local and systemic pathways. Fourth, the immunohistochemical findings were not validated using complementary molecular methods such as Western blotting and/or quantitative PCR. Although immunohistochemical H-score analysis provides semiquantitative information regarding the intensity and tissue distribution of immunoreactivity, it does not independently quantify total protein abundance or gene expression and cannot confirm the activation or inhibition of specific signaling pathways. Accordingly, these findings should be interpreted as associative tissue-level evidence rather than definitive molecular validation. Future studies combining immunohistochemistry with Western blotting and quantitative PCR are required to validate the observed changes in protein and transcript levels and strengthen the mechanistic interpretation of the findings. Fifth, bronchoalveolar lavage fluid (BALF) cytokine measurements, BALF inflammatory cell counts, and lung wet-to-dry weight ratio assessments were not performed. These parameters may provide additional quantitative information regarding pulmonary inflammatory cell recruitment and edema formation and should be considered in future studies. Finally, although intraperitoneal CCl_4_ administration is a well-established experimental model of pulmonary injury, it does not fully reproduce the multifactorial pathophysiology of human lung diseases associated with cigarette smoke, environmental pollutants, or respiratory infections. Future studies employing additional experimental models together with advanced molecular analyses are warranted to further elucidate the protective effects and underlying mechanisms of curcumin.

### 4.2. Translational Relevance

The present findings should be regarded as preclinical evidence obtained from a CCl_4_-induced lung injury model in male rats. Although oxidative stress, inflammation, fibrotic remodeling, and apoptosis-related processes are relevant to human pulmonary pathology, the experimental dose of curcumin and the systemic exposure achieved in this model cannot be directly extrapolated to humans. In particular, the limited oral bioavailability of conventional curcumin and the absence of plasma or lung tissue pharmacokinetic measurements preclude conclusions regarding whether comparable biologically active concentrations could be achieved clinically [10,36]. Furthermore, the present study does not establish a novel molecular mechanism or distinguish direct pulmonary effects from indirect effects mediated through the attenuation of hepatic and systemic injury. Therefore, the translational implications of these findings remain hypothesis-generating. Future studies should integrate pharmacokinetic and exposure–response assessments, evaluate bioavailability-enhanced curcumin formulations, and examine hepatic and pulmonary outcomes concurrently before the clinical relevance of these findings can be established.

## 5. Conclusions

The present study demonstrated that curcumin attenuated CCl_4_-induced lung injury in rats. CCl_4_ exposure caused pronounced pulmonary damage characterized by alveolar septal thickening, hemorrhage, vascular congestion, inflammatory cell infiltration, epithelial desquamation, vacuolization, and increased connective tissue deposition. These histopathological alterations were accompanied by increased MDA levels, reduced SOD activity, and higher TNF-α, IL-1β, TGF-β, and caspase-3 immunoreactivity, consistent with the involvement of oxidative stress, inflammation, fibrotic remodeling, and apoptosis-related processes in CCl_4_-induced lung toxicity.

Curcumin administration was associated with improved lung tissue morphology, reduced lipid peroxidation, increased antioxidant activity, and lower immunoreactivity for inflammatory, profibrotic, and apoptosis-related markers. Collectively, these findings support an attenuation of pulmonary injury associated with CCl_4_ exposure. However, because hepatic injury was not evaluated, the present data do not establish whether the pulmonary improvement resulted from direct actions of curcumin within the lung, indirect effects mediated by the attenuation of hepatic injury and systemic inflammation, or a combination of both. Concurrent assessment of hepatic and pulmonary outcomes in future studies is required to clarify the relative contributions of organ-specific and systemic mechanisms to these observations.

## Figures and Tables

**Table 1:** Histopathological scoring results obtained from lung tissues of the subjects.

Group	Vascular Congestion	Hemorrhage	Alveolar Septal Thickening	Inflammatory Cell Infiltration	Epithelial Desquamation	Alveolar/Septal Macrophagesfootnote	Vacuolization
Control	0 (0.0–1.0)	0 (0–0)	0.5 (0–1.0)	0.75 (0–1.0)	0 (0–1.0)	0 (0–1)	0 (0–0)
Curcumin	0 (0–0)	0 (0–0)	0.5 (0.5–0.5)	0.75 (0–1.0)	0 (0–1.0)	0 (0–1)	0.25 (0–0.5)
CCl_4_	2.75 * (2.0–3.0)	3.0 * (3.0–3.0)	3.0 * (3.0–3.0)	3.0 * (3.0–3.0)	1.75 * (1.5–2.0)	1.5 * (0–2.0)	3.0 * (3.0–3.0)
CCl_4_ + Cur	1.25 *^,†^ (1.0–2.0)	1.0 *^,†^ (1.0–1.5)	1.0 ^†^ (0.5–1.0)	1.0 ^††^ (1.0–1.0)	1.0 **^,†^ (0.5–2.0)	0 ^††^ (0–0)	0.75 ***^,††^ (0–1.5)

Data are presented as median (minimum–maximum). * *p* < 0.01 vs. control and curcumin groups; ** *p* < 0.05 vs. control and curcumin groups; *** *p* < 0.05 vs. control group; ^†^ *p* < 0.05 vs. CCl4 group; ^††^ *p* < 0.01 vs. CCl4 group.

**Table 2:** H-score values for TNF-α, IL-1β, TGF-β, and caspase-3 immunoreactivity in the lung tissues from all experimental groups.

Protein	Control	Curcumin	CCl_4_	CCl_4_ + Cur
TNF-α	62 (37–68)	63 (46–70)	220 * (188–269)	73 (58–91)
IL-1β	44.5 (36–63)	46 (31–58)	279 * (251–289)	63 (48–70)
TGF-β	22.5 (11–39)	22.5 (18–37)	274 * (189–291)	125 ** (76–152)
Caspase-3	39.5 (25–52)	46 (35–61)	260 * (224–299)	64.5 (38–74)

Values are presented as median (min–max). * Different from the control, curcumin, and CCl_4_ + Cur groups, ** Different from the control and curcumin groups. (*p* < 0.05 for all).

## Data Availability

The data supporting the findings of this study are available within the article. Further data are available from the corresponding author upon reasonable request.

## Abbreviations

The following abbreviations are used in this manuscript:

AP-1	Activator protein-1
Apaf-1	Apoptosis activation factor-1
CCl_4_	Carbon tetrachloride
COX-2	Cyclooxygenase-2
DAB	Diaminobenzidine
DISC	Death-inducing signaling complex
DNA	Deoxyribonucleic acid
ERK	Extracellular regulated kinase
GSH	Glutathione
GPx	Glutathione peroxidase
HIF-1	Hypoxia-inducible factor-1
IL-1β	Interleukin 1-beta
MAPK	Mitogen-activated protein kinase
MDA	Malondialdehyde
NADPH	Nicotinamide adenine dinucleotide phosphate
NF-κB	Nuclear factor kappa B
NLRP3	Nucleotide-binding and oligomerization (NOD) like receptor (NLR) containing pyrin domain3
ROS	Reactive oxygen species
SOD	Superoxide dismutase
TGF-β	Transforming growth factor-β
TNF-α	Tumor necrosis factor alpha
TNFR1	TNF-α receptor–1
TXNIP	Thioredoxin-interacting protein
